# Integration of Untargeted Metabolomics and Object-Oriented Data-Processing Protocols to Characterize Acerola Powder Composition as Functional Food Ingredient

**DOI:** 10.3390/antiox12071341

**Published:** 2023-06-25

**Authors:** Kewen Wang, Lu Mi, Xue Wang, Linyan Zhou, Zhenzhen Xu

**Affiliations:** 1 College of Food Science and Nutritional Engineering, China Agricultural University, Beijing 100083, China; wangkewen@cau.edu.cn (K.W.); milu@cau.edu.cn (L.M.); 2 Key Laboratory of Agro-Food Safety and Quality, Institute of Quality Standard & Testing Technology for Agro-Products, Ministry of Agriculture and Rural Affairs, Chinese Academy of Agricultural Sciences, Beijing 100081, China; woshiyangjiaming@sina.cn; 3 Faculty of Food Science and Engineering, Kunming University of Science and Technology, Kunming 650500, China; 20180012@kust.edu.cn

**Keywords:** acerola powder composition, functional food ingredients, metabolomics, antioxidant activity

## Abstract

Acerola powder has been experiencing a surge in demand as a functional food ingredient, particularly due to its usage in vitamin C supplements. However, limited research has been conducted on its other bioactive compounds. In this study, we employed metabolomics and object-oriented data-processing protocols to comprehensively characterize acerola powder. To ensure maximum coverage of metabolomics, we selected a 50% methanol aqueous solution as the extraction solvent and utilized the HSS T3 column for chromatography analysis. Through this approach, we successfully identified a total of 175 compounds in acerola powder, encompassing amino acids and peptides, polyphenols, organic acids, and various other compounds. Additionally, we measured the total phenolic content (TPC) and assessed the antioxidant activity of acerola powder. Furthermore, we analyzed the differential composition of acerola fruit and juice powder, identifying polyphenols and lipids as primary markers in fruit powder, while peptides emerged as key markers in juice powder. Notably, two specific peptides, Thr-Trp and Val-Tyr, were identified as antioxidant peptides. Overall, our study provides novel composition data for acerola powder, shedding light on its potential as a functional food ingredient. These findings contribute to the development and utilization of acerola powder in the formulation of functional food products.

## 1. Introduction

Functional food ingredients can provide us with health benefits over basic nutrition [1]. Tropical fruit, such as acerola, passion fruit, orange, and mango, as well as their products, are regarded as functional food ingredients for their bioactive molecules [2]. Among them, acerola relies on its high vitamin C content of 1000 to 4500 mg/100 g fresh fruit, which is around 50–100 times that of orange [3,4,5], and which has gradually attracted the attention of the public. Acerola can be eaten raw or processed into less perishable forms, such as juice and powder. Acerola powder has been displaying an increasing demand as a functional food ingredient, and the powder segment is dominating at a CAGR of 8.7% during 2019–2026 (https://straitsresearch.com/report/acerola-market/, accessed on 26 February 2023). A large part of this increase is due to the high usage of it in health and vitamin supplements, as well as its application in consumer and industrial products such as antioxidants. Some researchers have discovered that acerola powder possesses antioxidant and anti-inflammatory properties [6,7]. The existing research mainly attributes the above health benefit of acerola powder to natural vitamin C, while the other bioactive compounds in acerola powder is still unclear.

Compound identification is conducive to more sufficient descriptions of functional food ingredients, which is necessary to obtain scientific acceptance for functional foods [8]. Using liquid chromatography–tandem quadrupole time-of-flight mass spectrometry (LC−QTOF MS) to analyze untargeted metabolomics provides high-coverage features of food composition [9,10,11,12]. Processing and analyzing high-throughput metabolomics data is complicated, and multiple steps, including raw data processing, data cleaning, metabolite identification, statistical analysis, and even biological function exploration, are required [13]. Recently, the Tidymass project, depending on R packages, could provide a reproducible, comprehensive, and object-oriented computational framework [14]. The uniform data structure (object) of Tidymass offers a composition analysis of acerola powder in less time compared to other tools.

In this study, we obtained samples of acerola powder, specifically juice and fruit powder. Our research aimed to investigate the impact of different extraction solvents and chromatographic columns on the coverage of metabolomics analysis. We also utilized the object-oriented Tidymass project to analyze and characterize the detailed composition of acerola powder. Furthermore, we conducted assessments of the total phenolic content (TPC) and antioxidant activity of the acerola powder samples, using ABTS and DPPH assays. These measurements provided insights into the potential health benefits and antioxidant properties of acerola powder. Finally, we compared the differential composition of acerola powder derived from juice and fruit sources. This analysis allowed us to identify and understand the variations in composition between the two types of acerola powder, providing valuable information for further exploration and potential applications.

## 2. Materials and Methods

### 2.1. Chemicals and Reagents

Acetonitrile and methanol were purchased from Thermo Fisher Scientific (Waltham, MA, USA), and formic acid was purchased from DiKMA Technologies (Beijing, China). Water was generated by a Milli-Q Integral water purification system (Millipore Billerica, Billerica, MA, USA). Folin–Ciocalteu phenol reagent was purchased from Solarbio Life Sciences (Beijing, China). The antioxidant capacity assay kit with a Rapid ABTS method was purchased from Beyotime Biotechnology (Shanghai, China). The antioxidant capacity assay kit by DPPH assay was purchased from Dojindo Beijing Co., Ltd. (Beijing, China). The standards of vitamin C, p-coumaric acid, caffeic acid, trans-ferulic acid, kaempferol, and quercetin were purchased from Alta Scientific Co., Ltd. (Tianjin, China).

### 2.2. Extraction and Determination of Total Phenolic Content

TPC was measured with the Folin–Ciocalteu phenol reagent (F-C) according to the previous method described by Wang with some modifications [15]. Briefly, 2 mg of powder sample was dissolved in 1-mL 50% methanol aqueous solution, and extracted for 10 min by ultrasound. Then, 15-μL extraction, 65-μL water, 30-μL F-C reagent, and 80-μL NaCO3 (20%) were added successively to 96-well plates. After incubating for 30 min at 37 °C, the absorbance was measured using a microplate reader (Infinite M200 Pro, Tecan, Switzerland) at 765 nm. TPC results were expressed as milligram gallic acid equivalents per gram of powder sample (mg GAE/g powder).

### 2.3. Extraction and Determination of the Antioxidant Activity

A total of 1 mg powder sample was dissolved in 2 mL of 50% methanol aqueous solution, and extracted for 10 min by ultrasound. Then, the antioxidant activity of powder, based on the scavenging activity of ABTS and DPPH free radicals, was carried out by Total Antioxidant Capacity Assay Kit with a Rapid ABTS method (S0121, beyotime) and DPPH Antioxidant Assay Kit (D678-12, dojindo), respectively. The antioxidant activity was expressed as millimole Trolox equivalent antioxidant capacity per kilogram powder sample (mmol TEAC/kg powder).

### 2.4. Metabolomic Analysis

#### 2.4.1. Sample Collection and Pretreatment

The acerola powder was collected from five trusted local suppliers. The samples from suppliers A, D, and E were made from acerola juice and denoted as acerola juice powder, while the other samples (from suppliers B and C) were made from acerola fruit and denoted as acerola fruit powder. To investigate the effect of the extraction solvent and column on metabolomics coverage, acerola powder samples were extracted by two extraction solvents (50% methanol aqueous solution and methanol), separated by two chromatographic columns (HSS T3 and BEH Amide), totaling four methods, and analyzed via untargeted LC−QTOF MS. Specifically, the processing of acerola juice powder and acerola fruit powder is introduced in Figure S1. The physical and chemical indexes of samples from five local suppliers are shown in Table S1.

The pretreatment of samples was referred to a previous method with some modifications [16]. Briefly, the 1 g powder was accurately weighed, and placed in a 50 mL beaker with 10 mL 50% methanol aqueous solution or methanol. The sample was ultrasounded for 20 min and centrifugated at 9000 r/min for 5 min. The 8 mL supernatant was evaporated, and 1.5 mL 50% methanol aqueous solution or methanol was added to dissolve the residue before LC−QTOF MS analysis.

#### 2.4.2. Metabolomics Data Acquisition

The LC-QTOF MS acquisition was performed according to our previous study [15]. ACQUITY UPLC HSS T3 (1.8 μm, 2.1 × 100 mm) and BEH Amide (1.7 μm, 2.1 × 100 mm) were chosen as chromatographic columns. The column and autosampler were maintained at 40 °C and 4 °C, respectively. The flow rate and injection volume were 0.30 mL/min and 2 μL, respectively. Mobile phases A and B were 0.2% formic acid in the water and acetonitrile, respectively. The solvent gradient was: 0–11.50 min, 5−30% B; 11.50–11.51 min, 30–100% B; 11.51–15.00 min, 100% B; 15.00–15.01 min, 100–5% B; and 15.01–18.00 min, 5% B.

QTOF MS (TripleTOF 6600, USA) was applied for mass spectrometric analysis in both the positive and negative modes. The instrument was calibrated every 6 sample injections using an APCI calibration solution. The parameters of the TOF MS scan were set as follows: temperature, 500 °C; ion source gas 1, 50; ion source gas 2, 50; curtain gas, 25; ion spray voltage floating, 5.5 kV (−5.5 kV for negative mode); declustering potential, 60 V (−60 V for negative mode); collision energy, 10 eV (−10 eV for negative mode). The IDA with a cycle time of 545 ms was composed of a TOF MS scan (accumulation time, 50 ms; CE, 10 eV for positive mode and −10 eV for negative mode) and 15 dependent product ion scans (accumulation time, 30 ms each; CE, 35 eV for positive mode and −35 eV for negative mode) in the high sensitivity mode with dynamic background subtraction.

#### 2.4.3. Data Processing and Statistical Analysis

**Figure 1:**
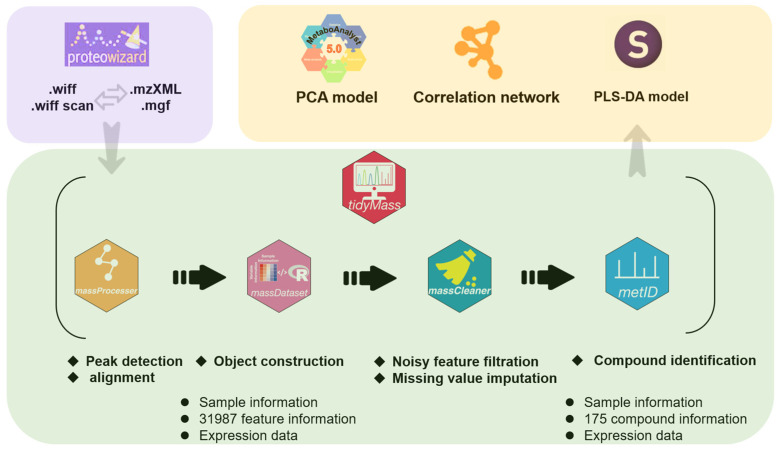
The object-oriented workflow of data processing and statistical analysis.

The object-oriented workflow of data processing and statistical analysis is shown in Figure 1. The acquisition data in .wiff and .wiff scan format was converted into data in .mzXML and .mgf format by ProteoWizard (version 3.0.22279) as MS and MS2 data for subsequent R analysis, respectively. Then, the converted data were processed by R (version 4.2.2) with Tidymass containing a series of packages [14]. First, the massprocesser package was applied to realize peak detection and alignment of raw MS data. The MS1 tolerance was 15 ppm in peak detection, the MS1 and MS2 tolerance was 10 ppm, and the retention time (RT) deviation was 15 s in peak alignment. Second, the massdataset package was applied to form the object containing sample information, feature information, and expression data. The data in .mgf format was also added to the object as MS2 data. Third, the masscleaner package was applied to filter noisy features and impute the missing value of the object. The features with detection rates reaching 80% in the QC group and 50% in at least one sample group were retained. The missing value is filled based on K-Nearest Neighbor. Fourth, the metid package was applied to execute compound identification by matching with public and in-house databases based on accurate mass (*m*/*z*), MS2 spectra, and/or RT, and the compounds with an annotation level of 1 and 2 were kept. The MS1, MS2, and RT tolerance were 25 and 30 ppm as well as 30 s in compound annotation. The final processed object was exported as a data frame containing sample information, compound information, and expression data.

Online platform Metaboanalyst (version 5.0) was applied to plot the principal component analysis (PCA) score plot using data matrices generated through the 175 identified compounds. At the same time, Cystoscope (version 3.9.0) was applied to plot the correlation network for each class of the 175 identified compounds (correlation coefficient > 0.5, *p*-value < 0.05). Additionally, SIMCA 14.2 was applied to construct the partial least squares discriminant analysis (PLS-DA) model and plot the biplot using data matrices generated through 175 identified compounds.

## 3. Results

### 3.1. Effects of Different Solvents and Columns on Composition Analysis of Acerola Powder

One of the primary parameters affecting metabolomics coverage is the polarity of the solvent [17]. Despite the different polarities of the solvents used, the acerola cherry powder was uniformly dispersed in the solvents and did not exhibit insolubility during the extraction process. In particular, all the acerola powder samples have good water solubility, indicating polar compounds dominate the samples. Considering the presence of abundant polar compounds in acerola powder, we chose two columns, HSS T3 and BEH Amide, which were excellent in polar compound separation. HSS T3 column belongs to the low-ligand-density C18 column and enables compounds to enter the pore structure more readily, providing balanced retention of polar and nonpolar compounds [18]. The BEH Amide column uses a trifunctionally bonded amide phase to enable a new dimension of separation, promoting exceptional retention of polar compounds spanning a wide range in polarity, structural moiety, and pKa depending on BEH particle technology [19].

**Figure 2:**
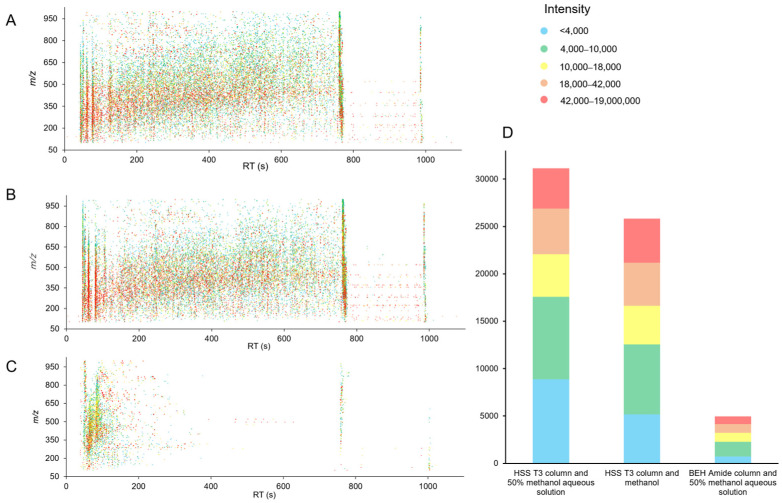
Bubble plots of acquired features under different extraction solvents and chromatographic columns in positive mode: (**A**), HSS T3 column, and 50% methanol aqueous solution. (**B**), HSS T3 column and methanol. (**C**), BEH Amide column and 50% methanol aqueous solution. Bubbles with different colors denote features of different *m*/*z* and RT. (**D**), Stacked columns showing the number of features with different intensities based on various extraction solvents and chromatographic columns.

Total ion chromatograms illustrate the change in total ion intensity over time in acerola powder samples (Figure S2). The stable and representative data acquisition could be applied to subsequent analysis. Each data point in Figure 2A–C represents a unique feature characterized by distinct *m*/*z* and RT values. To effectively showcase the number of features, we have included stacked columns indicating the number of features with different intensities based on various extraction solvents and chromatographic columns. In Figure 2D, we observed that when using the TSS T3 column, 50% aqueous solution resulted in the detection of a higher number of features, and a slightly greater abundance of high-level features compared to methanol. Consequently, the 50% methanol aqueous solution holds a slight advantage over methanol. The metabolomics coverage of plant material increases with the increasing solvent polarity [20], and the 50% methanol aqueous solution with higher polarity explains it. However, changing the BEH Amide column to the HSS T3 column with the same extraction solvent, the number of features in positive modes increases significantly and the proportion of high-intensity features improves (Figure 2A,C), indicating the enhancement of metabolomics coverage. Due to the low sensitiveness of the amide moiety, the BEH Amide column is less reactive to pH alteration of the mobile phase, and the reversible absorption of analytes to the column is limited [21], causing the reduction of features. The results in negative modes were identical.

**Figure 3:**
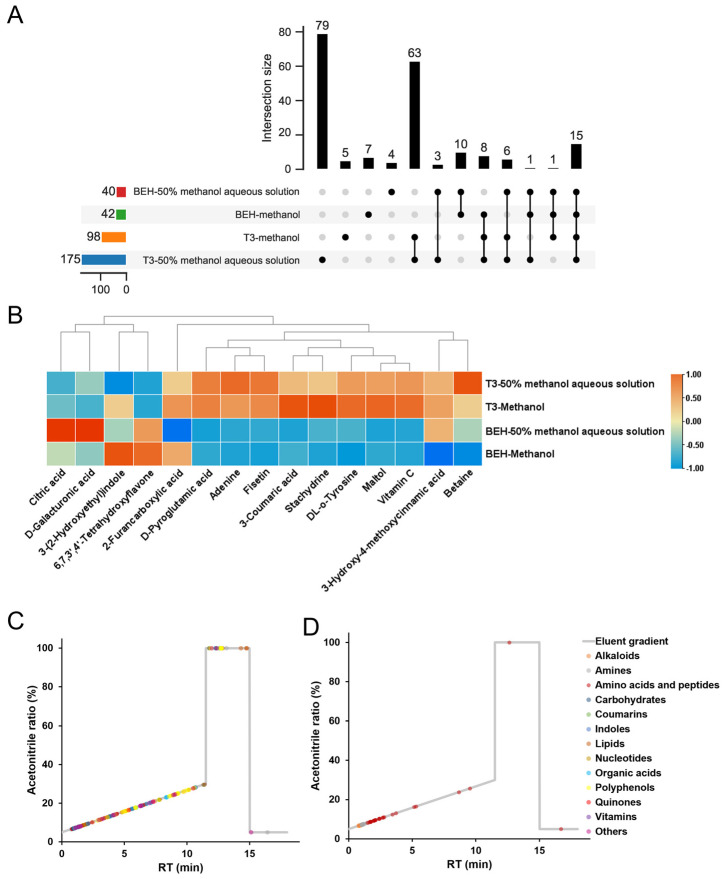
Effects of different extraction solvents and chromatographic columns on the quantities, intensity, and types of identified compounds in acerola powder samples. (**A**), the upset plot of the identified compound quantities in acerola powder samples. (**B**), the heatmap of peak area response of 15 compounds in acerola powder samples identified by all four methods. (**C**,**D**), the retention time (RT) and the corresponding mobile phase of identified compounds in acerola powder samples extracted by 50% methanol aqueous solution and separated by HSS T3 or BEH Amide, respectively.

The features were matched with public and in-house databases, and the identified compound quantities in acerola powder samples were displayed in the upset plot (Figure 3A). The pattern of identified compound quantities was consistent with that of the feature numbers. For the HSS T3 column, 50% methanol aqueous solution as the extraction solvent characterized more compounds compared to methanol as the extraction solvent. For the BEH Amide column, no matter which extraction solvent was used, the identified compounds were less than those detected by the HSS T3 column. By taking the intersection of these identified compounds, a total of 15 compounds can be identified in all four methods, and their average abundance in acerola powder samples detected by the four methods is shown in the heatmap (Figure 3B). The peak area of 12 compounds detected by the HSS T3 column was significantly higher than that detected by the BEH Amide column. Moreover, the RT and the corresponding mobile phase of the identified compounds were mapped. Various compounds mainly flowed out at the acetonitrile ratio of 5–30% using the HSS T3 column (Figure 3C), while compounds mainly flowed out at the acetonitrile ratio of 5–20% using the BEH Amide column (Figure 3D), which might produce stronger ion suppression.

The aforementioned results showed that 50% methanol aqueous solution and HSS T3 column not only maximize metabolomics coverage and enhance feature intensity but also improve both the quantity and intensity of identified compounds. Therefore, the subsequent composition analysis was generated from the identified compounds extracted by 50% methanol aqueous solution and separated by the HSS T3 column.

### 3.2. Composition Analysis of Acerola Powder

**Figure 4:**
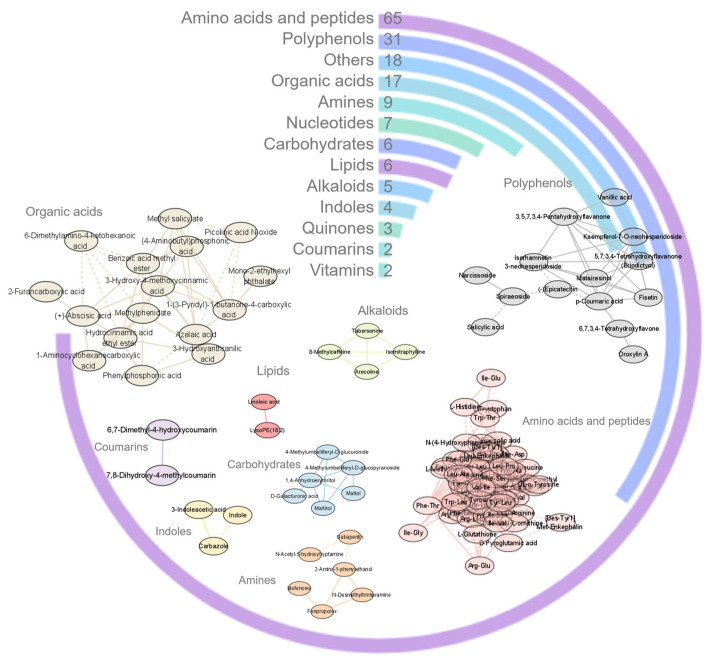
The exhibition of identified compounds in acerola powder samples. The circles show the classification and quantities of identified compounds in acerola powder samples. The networks show the intensity correlations of identified compounds in acerola powder samples. Solid lines show a positive correlation and dashed lines show a negative correlation. Only correlation with a correlation coefficient > 0.5 as well as a *p*-value < 0.05 is displayed.

The acerola powder samples extracted using a 50% methanol aqueous solution and separated by the HSS T3 column were subjected to further analysis. This analysis revealed the presence of a total of 175 compounds, encompassing various categories, including 65 amino acids and peptides, 31 polyphenols, 18 organic acids, 6 carbohydrates, 6 lipids, 2 vitamins, and 7 other categories of compounds (as shown in Figure 4 circle plot). To explore the relationships between these compounds, a correlation analysis was performed within each class of compounds. The results were visualized in network plots (Figure 4). The analysis indicated significant positive correlations (correlation coefficient > 0.5, *p*-value < 0.05) among compounds within the same class. This suggests that compounds within each class tend to exhibit consistent patterns in the acerola powder samples.

**Figure 5:**
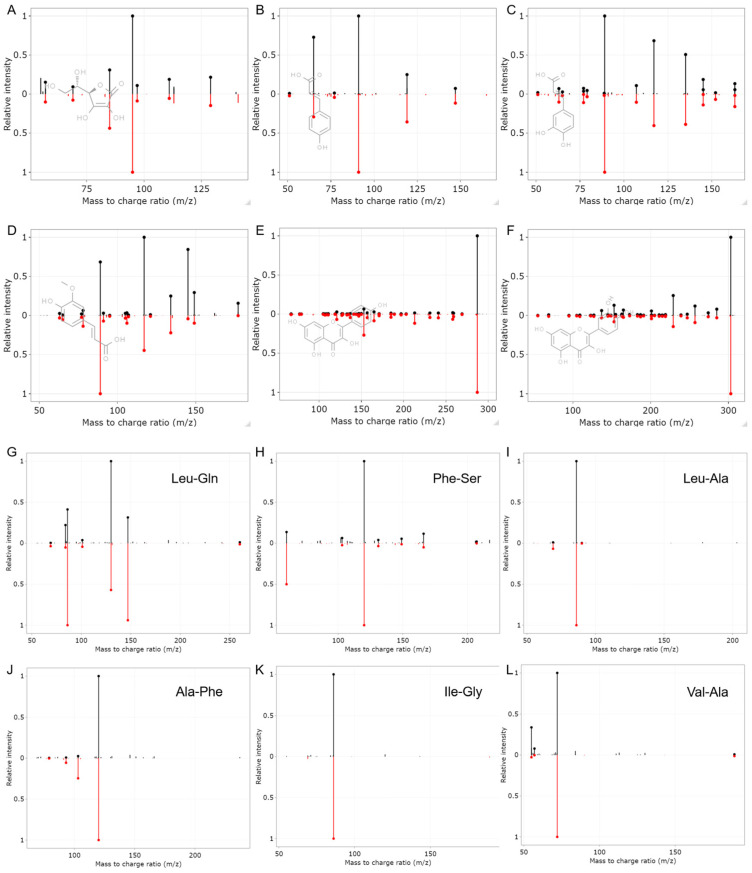
The mirror plots and structures of representative compounds with the annotation level of 1: (**A**), vitamin C, (**B**), p-coumaric acid, (**C**), caffeic acid, (**D**), trans-ferulic acid, (**E**), kaempferol, and (**F**), quercetin. (**G**–**L**), The mirror plots of representative peptides with the annotation level of 2.

Acerola is an acidic fruit, and the pH value varies from 3.60 to 3.70 [22], the acidity expressed in gram malic acid equivalent ranges from 1.04 to 1.87 [23]. Its acidity comes from organic acids and vitamin C. We observed that when it was powdered, the pH value is 2.80–3.60 (Table S1). The acerola powder displayed a lower pH value compared to acerola, which may be caused by the enrichment of organic acids and vitamin C during the spraying–drying process. In order of predominance, the organic acids in acerola are malic, citric, and tartaric acids [22]. The citric acids were retained in our acerola powder samples. Acerola is well-known for its high vitamin C content [24], and vitamin C in acerola powder samples is 17.0–19.2% (Table S1). Based on metabolomics, we have also detected vitamin C in all the acerola powder samples, which was matched to our in-house database with the annotation level of 1 (Figure 5A). Additionally, vitamin B is also recorded in acerola, such as vitamin B6 [5]. Vitamin B6 was identified in all the acerola powder samples. The aforementioned results indicated that the process of acerola powder from acerola could maintain previously reported vital nutrients, such as organic acids as well as vitamins.

Another kind of component in acerola is phenolic compounds [25,26]. Phenolic compounds including benzoic acid derivatives (syringic acid, salicylic acid, etc.), phenylpropanoids (p-coumaric acid, caffeic acid, trans-ferulic acid, etc.), and flavonoids (kaempferol, quercetin, etc.) were identified in our acerola powder samples (Figure 5B–F). Previous studies also focused on abundant amino acids in acerola [16]. Interestingly, in addition to various amino acids, peptides were also identified in acerola powder samples. The mirror plots of representative peptides with an annotation level of 2 are shown in Figure 5G–L. On one hand, the peptides may naturally occur in acerola and go straight into the acerola powder. Fruit and vegetables and their products have low protein content, and acerola has even lower protein content ranging from 0.21% to 1.2% [27]. However, an increasing number of studies showed fruit and its products may be an important source of peptides, such as Tyr-isoLeu-Thr-Thr in organic tomato samples [28], 7 tripeptides in orange juice [29], and Arg-Thr-Asp in lemon juice [30]. On the other hand, it is worth noting that peptides or proteins in acerola powder may potentially be influenced by processing excipients used during the manufacturing process. Consequently, the exact source of the acerola powder peptides remains uncertain. Nevertheless, the discovery of these peptides in acerola powder represents a significant contribution to our understanding of the peptide composition within the fruit and its derived products.

Based on the identification of 175 compounds, the PCA analysis (Figure S3) revealed differences among acerola powder samples from five different suppliers. It is worth noting that the acerola powder samples from suppliers A, D, and E were derived from acerola juice, while the samples from suppliers B and C were obtained from acerola fruit. The disparity in processing primarily lies in the sample form before spray drying (Figure S1). These distinctions were reflected in principal component 2, with the juice powder samples positioned in the positive direction and the fruit powder samples positioned in the negative direction of principal component 2.

### 3.3. Differential Analysis of Acerola Juice Powder and Fruit Powder

**Figure 6:**
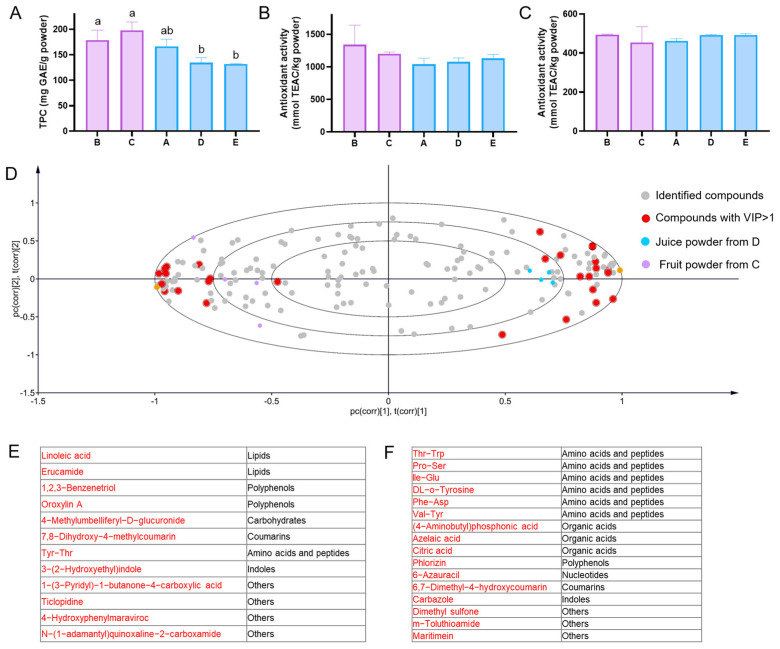
The analysis of quality and differential compounds in acerola juice powder and acerola fruit powder. (**A**), TPC in acerola powder samples. (**B**,**C**), antioxidant activity measured by assays of ABTS and DPPH in acerola powder samples. (**D**), the biplot of the PLS-DA model of peak area response in acerola powder samples. Data matrices for all samples were generated through 175 identified compounds. (**E**,**F**), the compounds with variable importance in projection (VIP) higher than 1 on the left and right of the biplot represent markers in acerola fruit and juice powder samples, respectively. Different letters of the same index represent significant differences (*p*-value < 0.05).

Acerola is famous for its high-level vitamin C as well as phytochemicals such as polyphenols [16]. Recently, much attention has been paid to the content in TPC for their antioxidant properties in acerola powder [31]. The TPC and antioxidant activity (ABTS and DPPH) of acerola powder from five different suppliers are shown in Figure 6A–C. The TPC of acerola juice powder and fruit powder was between 120 and 210 mg GAE/g powder, which was consistent with the 203.8 mg GAE/g powder of TPC reported in previous literature [31]. Furthermore, we found that TPC is markedly different in acerola juice powder and fruit powder, and acerola fruit powder contained significantly higher TPC. The antioxidant activity measured by assays of ABTS and DPPH is 950–1700 and 450–510 mmol TEAC/kg powder in acerola powder, respectively. The antioxidant activity of acerola powder samples has not been previously studied, while the antioxidant activity of acerola pulp has been extensively studied and reported. The documented values for the antioxidant activity of acerola pulp range from 30 to 90 mmol TEAC/kg pulp when measured using the ABTS assay, and from 40 to 160 mmol TEAC/kg pulp when measured using the DPPH assay [16,25]. The data from suppliers show 1 kg of powder is made from around 10 kg of fresh acerola (edible part), and the antioxidant activity result measured by assays of ABTS and DPPH in our study is consistent with the previous results. Moreover, the results of antioxidant activity measured by assays of ABTS and DPPH showed no significant distinction in acerola juice and fruit powder.

Aligned with the limitations observed in previous targeted studies, the outcomes pertaining to traditional quality assessment of acerola juice powder and fruit powder were insufficient in demonstrating significant differences. Therefore, an untargeted analysis approach leveraging high-throughput technology, in conjunction with the variable importance in projection (VIP) value in the PLS-DA model, was employed to identify distinctive compounds between acerola juice and fruit powder. The relationship between samples and the identified compounds is illustrated in Figure 6D through a biplot. The fruit powder samples (depicted by purple circles) are positioned on the left side, while the juice powder samples (depicted by blue circles) are situated on the right side of the biplot. The red circles represent compounds with VIP scores greater than 1. Notably, the red circles on the left side correspond to compounds that exhibit significantly higher levels in fruit powder samples, particularly lipids, polyphenols, and sugars (Figure 6E). This finding is consistent with the TPC, which suggests that acerola fruit powder contains a higher concentration of polyphenols compared to acerola juice powder.

The red circles on the right represent compounds with significantly higher content in juice powder samples, mainly amino acids and peptides, and organic acids (Figure 6F). There are 5 peptides in acerola fruit powder, among which Thr-Trp and Val-Tyr were reported as antioxidant peptides. The peptide Thr-Trp showed antioxidant activity with an IC50 value of 4.8 mM [32]. Val-Tyr, which is also significantly elevated in acerola juice powders, could inhibit autoxidation of soybean oil-in-water emulsions, indicating its antioxidative property [33], and it was proven as an antioxidant peptide by ABTS assay [33]. Although there was no significant difference in the total antioxidant activity between acerola fruit and juice powder, the antioxidant peptides in acerola juice powder should be further investigated.

## 4. Discussion

Acerola powder has experienced a significant surge in demand as a functional food ingredient, primarily attributed to its widespread use in health and vitamin C supplements. However, there remains a dearth of research concerning other compounds present in acerola powder. In this study, we undertook a novel approach by employing untargeted metabolomics and object-oriented data-processing protocols to thoroughly characterize the functional properties of acerola powder for the first time.

To ensure comprehensive coverage of metabolites in the acerola powder sample, we conducted optimization experiments on the chromatographic columns and extraction solvents. Following rigorous testing, we determined that the HSS T3 column exhibited the highest performance as the chromatographic column, while a 50% methanol aqueous solution proved to be the optimal extraction solvent. These parameters were chosen to maximize the detection and identification of metabolites present in the acerola powder sample. We identified a total of 175 compounds within the acerola powder sample. These compounds encompassed a diverse range of categories, including 65 amino acids and peptides, 31 polyphenols, 18 organic acids, 6 carbohydrates, 6 lipids, 2 vitamins, and 7 other categories of compounds. This comprehensive profiling of the acerola powder allowed us to gain valuable insights into its chemical composition and potential health benefits for the first time.

The PCA analysis conducted on the 175 identified compounds revealed notable differences between acerola fruit and juice powder. This indicates that the composition of acerola fruit and juice powder varies. TPC was found to differ significantly between acerola juice and fruit powder, with acerola fruit powder displaying significantly higher TPC. This observation aligns with the higher levels of polyphenols detected in the acerola fruit powder samples. On the other hand, amino acids, peptides, and organic acids exhibited higher content in the juice powder samples. Among them, five peptides attracted our interest. Notably, Thr-Trp and Val-Tyr have been reported as antioxidant peptides. Although there was no significant difference in the total antioxidant activity between acerola fruit and juice powder, the presence of antioxidant peptides in acerola juice powder warrants further investigation. Overall, these findings suggest that acerola fruit and juice powder have distinct compositions, with variations in TPC, polyphenols, amino acids, peptides, and organic acids. The identification of antioxidant peptides in acerola juice powder highlights a potential area for future research and exploration of acerola powder.

## 5. Conclusions

In summary, our study aimed to maximize the coverage of metabolomics in acerola powder by utilizing a 50% methanol aqueous solution as the extraction solvent and the HSS T3 column for chromatography analysis. By employing object-oriented data-processing protocols, we successfully identified a total of 175 compounds in acerola powder. These compounds included 65 amino acids and peptides, 31 polyphenols, 18 organic acids, 6 carbohydrates, 6 lipids, and 2 vitamins. Furthermore, we conducted assessments of total TPC and antioxidant activity (measured by ABTS and DPPH assays) in acerola powder. Our findings revealed that acerola fruit powder exhibited significantly higher TPC compared to acerola juice powder. Additionally, we compared the differential composition of acerola powder derived from fruit and juice sources. The markers distinguishing acerola fruit powder were primarily polyphenols and lipids, while peptides emerged as key markers in acerola juice powder. Notably, two peptides, Thr-Trp and Val-Tyr, were identified as antioxidant peptides. Overall, our study provides valuable composition data for acerola powder, which has not been extensively explored previously, particularly in the context of developing functional food ingredients. These findings contribute to a better understanding of the potential benefits and applications of acerola powder in the field of functional foods.

## Data Availability

The data presented in this study are available on request from the corresponding author.

## Supplementary Materials

The following supporting information can be downloaded at: https://www.mdpi.com/article/10.3390/antiox12071341/s1, Figure S1: The processing of acerola juice powder and acerola fruit powder. The icons used in this figure are from www.flaticon.com; Figure S2: Total ion chromatograms of acerola powder samples extracted by different solvents and separated by different columns in positive and negative modes; Figure S3: PCA score scatter plot of peak area response for the acerola powder samples from five factories. Data matrices for all samples were generated through the 175 identified compounds; Table S1: Type examination of acerola powder from five factories.
