# Supplementary material for: Integration of Untargeted Metabolomics and Object-Oriented Data-Processing Protocols to Characterize Acerola Powder Composition as Functional Food Ingredient
Source: Antioxidants (Basel). 2023 Jun 25;12(7):1341. doi: 10.3390/antiox12071341 (PMC10376614; doi:10.3390/antiox12071341)
Supplement: Supplementary file 1 [file antioxidants-12-01341-s001.zip › Figures S1-3.pdf]

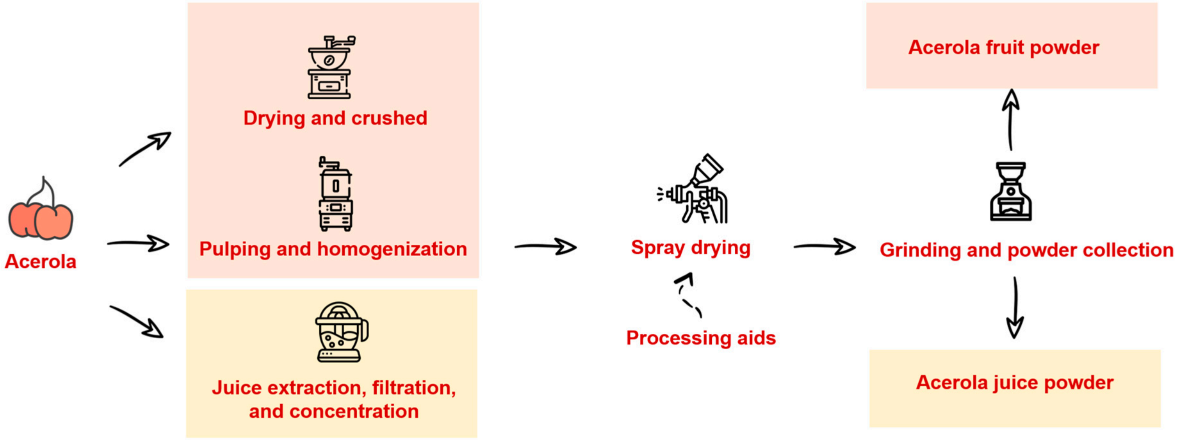

Figure S1. The processing of acerola juice powder and acerola fruit powder. The icons used in this figure are from www.flaticon.com.

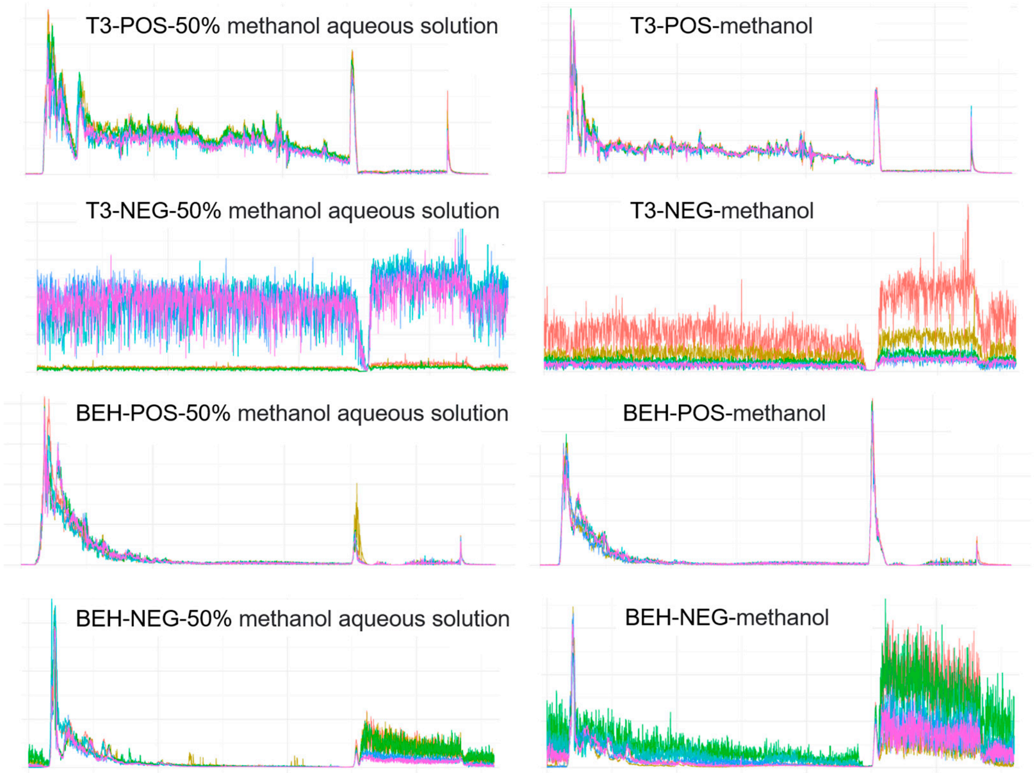

Figure S2. Total ion chromatograms of acerola powder samples extracted by different solvents and separated by different columns in positive and negative modes.

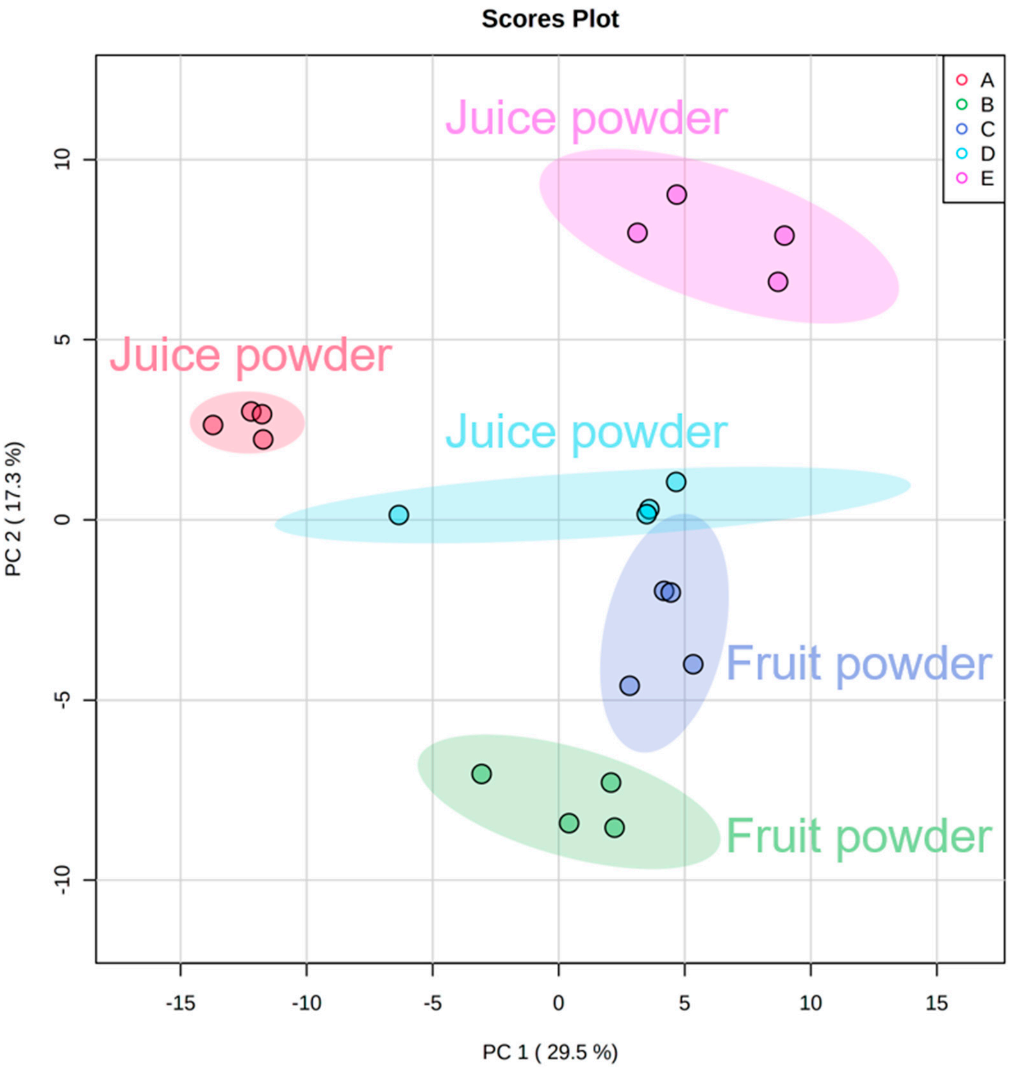

Figure S3. PCA score scatter plot of peak area response for the acerola powder samples from 5 factories. Data matrices for all samples were generated through the 175 identified compounds.
